# Gemcitabine/cisplatin versus 5-fluorouracil/mitomycin C chemoradiotherapy in locally advanced pancreatic cancer: a retrospective analysis of 93 patients

**DOI:** 10.1186/1748-717X-6-88

**Published:** 2011-07-27

**Authors:** Thomas B Brunner, Rolf Sauer, Rainer Fietkau

**Affiliations:** 1 Radiation Oncology of the Friedrich-Alexander University of Erlangen-Nuremberg, Universitätsstraße 22, 91054 Erlangen, Germany; 2 Gray Institute for Radiation Oncology and Biology, University of Oxford, Roosevelt Drive, Oxford OX3 7DQ, UK

**Keywords:** Pancreatic cancer, chemoradiotherapy, gemcitabine, 5-fluorouracil

## Abstract

**Background:**

Despite of a growing number of gemcitabine based chemoradiotherapy studies in locally advanced pancreatic cancer (LAPC), 5-fluorouracil based regimens are still regarded to be standard and the debate of superiority between the two drugs is going on. The aim of this retrospective analysis was to evaluate the effect of two concurrent chemoradiotherapy regimens using 5-fluorouracil or gemcitabine to compare their effect and tolerance.

**Methods:**

We have performed a single centre retrospective analysis of 93 patients treated with conventionally fractionated radiotherapy of 55.8 Gray using either concurrent 5-fluorouracil, 1 g/m² on days 1-5 and 29-33 of radiotherapy and 10 mg/m² of mitomycin C on day 1, 29 of radiotherapy (FM group, 35 patients) versus gemcitabine (300 mg/m²) and cisplatin, (30 mg/m²) on days 1, 8, 22, and 29 (GC group, 58 patients). Primary endpoint was the median overall survival (OS) rate.

**Results:**

The median OS rate was 12.7 months in the GC group and 9.7 months in the FM group. The 1-year OS rate was 53% versus 40%, respectively (p = 0.009). GC led to more grade 3 leukocytopenia and thrombocytopenia than FM, but not to more grade 4 myelosuppression. Thrombocytopenia was the most frequently observed grade 4 toxicity in both groups (11% after FM versus 12% after GC). No grade 3/4 febrile neutropenia was observed. Grade 3 nausea was more common in the FM group (20% versus 9%) and grade 4 nausea was observed in one patient per group only.

**Conclusions:**

GC was superior to FM for overall survival and both regimens were similar in terms of tolerance. We conclude that GC leads to encouraging results and that the use of FM for chemoradiotherapy in LAPC cannot be recommended without concerns.

## Background

Pancreatic ductal adenocarcinoma (PDAC), commonly known as pancreatic cancer, is the 10^th ^most common cancer type with an incidence of 10/100,000 but highly lethal (> 95%) and this is reflected by the fact that it is ranking as the 5^th ^most lethal cancer in absolute patient numbers after lung, colorectal, breast and prostate cancer [1,2]. Due to the declines in lethality in other major cancers, pancreatic cancer is predicted to become the fourth cause of cancer death in Europe [2]. Dramatic progress was made during the past years to better understand the biology of this disease (reviewed in [3]). Only 10-20% of the patients have resectable tumours at diagnosis and resection is a prerequisite for cure but even with adjuvant therapy median overall survival of resected patients is still as low as 20% after 5 years in randomised phase III studies (reviewed in [4]). The large majority (> 80%) of patients with non-resectable disease at diagnosis can be subdivided into metastatic and locally advanced PDAC (LAPC) with both stages being about equally frequent. Compared with metastatic disease patients with LAPC have a better prognosis and - though often grouped together with metastatic disease not separated in randomised phase III trials - patients with LAPC should be separated from patients with metastatic disease.

Chemotherapy is an essential element in the treatment of LAPC to fight the high tendency of distant spread. But the combination of systemic with local treatment prolonged survival in a number of recent studies [5,6] compared with systemic therapy only. Of note, secondary resection after CRT was reported in a systematic review and meta-analysis in 1/3 of the patients leading to a median overall survival (mOS) rate of 20.5 months which is equally good as after primary resection [7] and downstaging was also described [8]. On the other hand, the inferiority of chemoradiotherapy (CRT) vs chemotherapy in a recent French trial [9] can most likely be attributed to inadequate technique and quality of chemoradiotherapy highlighting the complexities of CRT for PDAC [10]. Of note, 60 Gy were delivered in 2 Gy fractions to both the primary tumour and the elective lymphatics resulting in large planning target volumes (PTV) as 2 cm expansion margins were used from the clinical target volumes. Also, the FFCD-SFRO trial [9] is the only randomised phase III CRT trial using 5-fluorouracil (5-FU)/Cisplatin as concurrent chemotherapeutic agents and this resulted in a very high rate of grade 3/4 toxicity for the adjuvant chemotherapy and prevented maintenance chemotherapy. Commonly, the combination of a fluoropyrimidine with radiotherapy is regarded to be the standard of care for CRT [4] but a substantial number of gemcitabine based CRT trials was reported with encouraging results such as in the ECOG-4201 trial [6]. The latter trial used IMRT together with 600 mg/m^2 ^gemcitabine weekly, a relatively high dose, resulting in a high rate of grade 3/4 toxicity.

The rationale for preferring gemcitabine over 5-FU in CRT regimens is its hypothesised superiority both, locally and systemically: in metastatic disease gemcitabine was able to prolong survival and to lead to higher clinical benefit compared to 5-FU [11]. For the local effect when used with radiotherapy, gemcitabine is predicted to lead to higher tumour cytotoxicity than 5-FU because it is one of the most potent radiosensitising chemotherapeutic agents [12]. Gemcitabine is an S-phase specific deoxycytidine analogue. It acts via competitive incorporation of dFdCTP and dCTP into DNA and results in DNA fragmentation and subsequent cell death. Furthermore, gemcitabine interferes with ribonucleotide reductase which is thought to have an impact on cell death by affecting DNA repair. Also, specific single-nucleotide polymorphisms in DNA the repair damage genes ATM, Chek1 and ATR were found to be significantly associated with OS after gemcitabine CRT especially when analysed for the combined effect of all three genes [13]. In line with these observations, gemcitabine containing schedules were described to achieve a higher rate of pathologic response compared to 5-FU based protocols [14]. The combination of gemcitabine with 5-FU or capecitabine which is commonly used as a chemotherapy combination was found to be too toxic for CRT in LAPC especially in terms of elevated gastrointestinal toxicity [15]. Therefore we decided a different chemotherapeutic combination, gemcitabine and cisplatin, which had been investigated both preclinically and clinically: the synergism between the two drugs is attributed mainly to an increase in platinum-DNA adduct formation which is possibly related to changes in DNA due to dFdC incorporation into the DNA [16-18]. The combination of the two drugs is clinically in use mainly in ovarian, non-small cell lung and pancreatic cancer and has been more effective than gemcitabine only in metastatic and locally advanced PDAC in the group of patients with good performance status [19].

Despite of this rationale, gemcitabine initially was difficult to be combined with radiotherapy due to its acute toxicity profile depending profoundly on the absolute radiotherapy treatment volume [18,20]. This potential dangerous effect can now be more easily counter-balanced with highly conformal treatment planning and the use of IMRT/IGRT thereby increasing the tolerance of gemcitabine based CRT [21]. In this analysis we compare the outcome and the toxicity of two CRT regimens in 93 patients with LAPC treated at our centre: One regimen was 5-FU/Mitomycin C (FM), the other gemcitabine/cisplatin (GC) given concurrently with radiotherapy. These two regimens have not been compared in the literature up to now but they both have been used in a number of trials in PDAC and other upper GI tumours [22-25]. We report superior OS of the GC regimen with comparable high grade toxicity (grade 4 haematologic and grade 3/4 non-haematologic disease).

## Methods

### Patient population

This is a retrospective study identifying all patients treated at the University Hospitals of Erlangen with chemoradiotherapy. Patients were identified by reviewing the tumour board minutes and the departmental minutes of Radiation Oncology. Patients with locally advanced pancreatic carcinoma (LAPC) were selected for primary CRT at our local tumour board. The following eligibility criteria were used: Histological proof of ductal adenocarcinoma prior to CRT. In general, LAPC was defined along the lines of the Practice Guidelines in Oncology™ of the National Comprehensive Cancer Network [26]: A minimal Karnofsky performance score ≥60% was required and pretherapeutic laboratory requirements for chemotherapy were: leukocyte count ≥4000/μL, platelet count ≥100,000/μL, bilirubin < 2.0 mg/dL, and a creatinine clearance ≥60 mL/min. Echocardiography was performed to ensure that prehydration before cisplatin chemotherapy was tolerable. Jaundiced patients underwent bile duct stenting prior to therapy. Patients being treated with either FM or GC chemotherapy were eligible. These two schedules were used almost exclusively in our institution. Choice between FM and GC is explained below.

### Treatment

Radiation treatment planning was performed as described elsewhere in detail [27]. Briefly, 3-D conformal treatment planning was applied based on IV and oral contrast enhanced planning CT scans. PTV_5040 (planning target volume) comprised the primary tumour (GTV) and elective lymphatic nodes and PTV_5580 comprised the GTV with margins only. Elective nodes treated in pancreatic head and body tumours were the regions 8, 9, 12, 13, 14, 16a2, 16b1, 17, and 18 to the right of the left edge of the aorta according to the Japanese Gastric Cancer Association [28]. The total PTV_5040 volume was not allowed to be larger than 800 mL. Conventional fractionation with single doses of 1.8 Gy was used. The dose constraints for the organs at risk were for the liver V30 < 50%, for the ipsilateral kidney V20 < 50% and for the contralateral kidney V20 < 30%, and D_max _to the spinal cord < 40 Gy.

**Figure 1:**
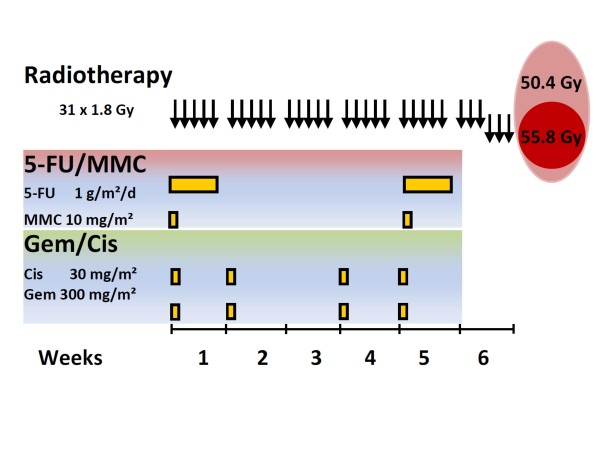
**Schedule of two chemoradiotherapy treatment schedules (gemcitabine/cisplatin versus 5-fluorouracil/mitomycin C**. Combination of radiotherapy with 5-fluorouracil and mitomycin C (FM) in the upper panel versus gemcitabine and cisplatin (GC) in the lower panel. Each arrow in the top panel corresponds to one daily fraction of radiotherapy. The last three arrows represent a boost restricted to the tumour (= Planning target volume, PTV5580) whereas the rest of the fractions included additionally the regional lymphatics (PTV5040) and this is illustrated in the right hand Euler diagram. Abbreviations: 5-FU = 5-fluorouracil; Cis = cisplatin; Gem = gemcitabine; Gy = Gray; MMC = Mitomycin C.

For the 5-fluorouracil and Mitomycin C regimen (FM), 5-FU was given as 24 h continuous infusion of 1000 mg/m2/day on days 1-5 and days 29-33. Mitomycin C was given as an IV bolus injection (10 mg/m2) on days 1 and 29 (Figure 1). For the gemcitabine/Cisplatin (GC) regimen, 300 mg/m2 gemcitabine and 30 mg/m2 cisplatin were given intravenously on days 1, 8, 22 and 29 being the first day of radiotherapy in weeks 1, 2, 4 and 5. Gemcitabine was given first followed immediately by Cisplatin which was given < 1 hour prior to radiotherapy. Supportive therapy comprised gastric acid protection during and at least 3 months after therapy, antiemetic therapy, and nutritional support which in most patients was given as supportive parenteral feeding as required. There was a gradual change in the institution from FM to GC after the completion of a phase I study on concurrent GC chemoradiotherapy [29]. After chemoradiotherapy some patients had additive gemcitabine chemotherapy (1000 mg/kg; d1, 8, 15, q29d) which was given at the discretion of the treating physician.

### Efficacy, treatment evaluation and statistical analysis

Follow up examinations were performed six weeks after the end of treatment and then every 3 months for the first 2 years after chemoradiotherapy and thereafter every 6 months for at least 3 years. In addition to physical examination, laboratory tests (full blood cell counts, biochemistry including liver and kidney function tests and CA-19-9), and ultrasound of the abdomen. CT abdomen and a chest X-ray or a CT abdomen/chest were performed every 6 months. Statistical data analysis was done with the software IBM Statistical Package for the Social Sciences^®^, version 19.0. Kaplan-Meier plots were calculated for analysis of survival. Survival was calculated from date of diagnosis to date of death or date of last contact. Pair wise log-rank test was employed for comparison of the differences in survival in subgroups of patients. The RTOG toxicity criteria [30], the LENT-SOMA criteria [31] (side effects of radiotherapy) and the CTCAE v3.0 toxicity criteria of the NCI (haematological side effects) were used to classify acute and chronic treatment-related side effects. The treatment was in accordance with the ethical standards of the local committee on human experimentation and with the Helsinki Declaration of 1975, as revised in 2000, and all patients provided informed consent before therapy.

## Results

### Patient characteristics

Ninety-three patients from our centre were treated with either FM or with GC chemoradiotherapy. The median follow-up time at analysis was 11.7 months. At the time of analysis 6 patients were alive (6%). The baseline patient characteristics are summarised in Table 1. The majority of the patients were diagnosed with cT4 tumours (52%). In both arms, 91% of the patients had an ECOG performance status of at least 2, respectively. All patients had ductal adenocarcinoma of the pancreas as diagnosed by biopsy or laparoscopically during an attempt of resection. Reasons for non-resectability were vascular involvement in most patients or nodal disease as diagnosed on contrast enhanced computed tomography.

**Table 1:** Patient characteristics

		5-FU, Mitomycin C		Gemcitabine, Cisplatin	
		patients	%	patients	%
All patients		35		58	
Age	Median (Range)	63 (37 - 75)		63 (35 - 76)	
Gender	Male	23	66	36	62
	Female	12	34	22	38
Surgery	No resection	26	74	41	71
	resection	9	26	15	29
Tumour location	Head	25	71	40	69
	Head/Body	3	9	5	9
	Body	5	14	9	16
	Body and tail	1	3	3	5
	tail	-	-	1	2
cT 1997	1	1	3	2	3
	2	8	23	8	14
	3	8	23	16	28
	4	18	51	32	55
cN 1997	0	15	43	27	47
	1	20	57	31	53
UICC 1997	I	4	11	7	12
	II	3	9	7	12
	III	9	26	12	20
	IVa	17	49	31	53
	IVb	2	6	1	1
Grading	1	3	9	3	5
	2	15	43	28	48
	3	5	14	16	28
	4	0	-	0	-

*Abbreviations: *R0 = clear resection, R1 = positive margin, RX = resection margin uncertain.

### Treatment and outcome

The median duration of radiotherapy was 43 days (SD 4.8 days) in all patients (FM, 43 days, SD 5.2 days; GC, 42 days, SD 4.9 days). In the FM group and in the GC group the median total doses to the PTV_5040 (primary and lymphatics) were 50.4 Gy (range 28.8 - 50.8 Gy; 41.4 - 55.8 Gy) and the cumulative doses to the PTV_5580 (GTV and margin) were 55.8 Gy (28.8 - 57.6 Gy; 41.4 - 59.4 Gy), respectively. Radiotherapy was not completed in four patients: two patients treated with FM developed distant metastasis during treatment (total dose 28.80 and 46.80 Gy), one patient with GC received a total dose of only 41.40 Gy due to decreasing performance status and 1 patient treated with GC could not be fully treated due to cholangitis after having reached a total dose of 50.4 Gy.

**Figure 2:**
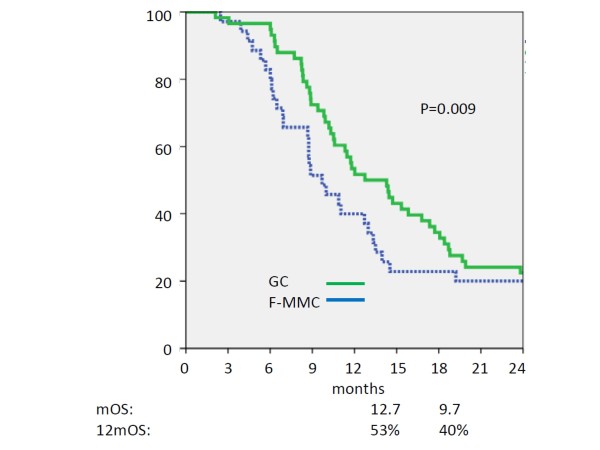
**Kaplan-Meier plot of overall survival of patients**. Concurrent chemoradiotherapy with gemcitabine/cisplatin (green solid line, n = 58) versus 5-fluorouracil/mitomycin C (blue dotted line, n = 35). Y-axis = percentage of patients surviving. Median overall survival time 12.7 vs 9.7 months; 1 year overall survival rate: 53% vs 40%.

Median overall survival time for all 93 patients was 11.5 months and 12 month overall survival rate was 48%. At analysis four patients were alive in the FM group and 2 in the GC group. Median overall survival for patients treated with FM was 9.7 months and 12.7 months for patients treated with GC (Figure 2). One-year overall survival rates were 40% in FM and 53% in GC treated patients and this difference was statistically significant (p = 0.009). Survival of 36 patients who had additive chemotherapy after radiotherapy was not statistically longer than that of the 57 patients without (p = 0.24). The vast majority of the patients died from metastatic disease. There was neither a statistically significant correlation between the use of additive chemotherapy, the dose intensity of additive chemotherapy and ECOG performance status nor between additive chemotherapy and the type of chemoradiotherapy (GC vs FM). Patients with FM and GC had a median of 7 cycles of gemcitabine.

### Chemotherapy dose reductions and tolerance results

As for the concurrent chemotherapy with radiotherapy, GC dose reductions were necessary in 22% of the patients. A dose reduction of FM was necessary in 14% of the patients. The main reasons for treatment delay or dose reduction of simultaneous chemotherapy were leukocytopenia, thromocytopenia or both combined for both treatment groups.

The acute toxicities during chemoradiotherapy according to the NCI-CTC criteria are shown in Table 2. The GC regimen led to more grade 3 leukocytopenia and thrombocytopenia than the FM regimen, but not to more frequent grade 4 myelosuppression. Combined grade 3/4 leukocytopenia of FM and GC were 37% and 48% respectively. No grade 3 or grade 4 febrile neutropenia was observed. Platelets count reduction was the most relevant grade 4 toxicity for both regimens at 11% and 12% respectively necessitating platelet prophylactic transfusions but no acute bleeding episodes were observed for both chemotherapy schedules. Combined grade 3/4 thrombocytopenia was 20% vs 36%, respectively in the FM and GC groups. Grade 3 upper gastrointestinal (GI) tract toxicity was more frequent with the FM regimen, again with no obvious difference for grade 4 upper GI toxicity being a rare event. Combined higher grade (3 or 4) nausea and vomiting were 37% vs 18% in FM and GC groups respectively. The median body mass index (BMI) was reduced by 1.0 kg/m^2 ^(standard deviation 0.98 kg/m^2^, range 0 - 4.4 kg/m^2^) in the GC group and 0.9 kg/m^2 ^(standard deviation 1.3 kg/m^2^, range -5.5 - 2.9 kg/m^2^) in the FM group at the nadir of the weight. For the 10 patients with a BMI < 20 kg/m^2 ^the median weight loss was 0.8 kg/m^2 ^(standard deviation 0.5 kg/m^2^, range 0 - 1.8 kg/m^2^). We also analysed long-term toxicity: although no patients had hepatotoxicity or renal toxicity during follow up, one patient in the GC group had a duodenal bleeding from an ulcer four months after the end of therapy which was fatal. Proton pump inhibitor treatment had been discontinued after treatment despite of being prescribed in the end-of-treatment letter.

**Table 2:** Acute toxicity of chemoradiotherapy according to CTC-NCI criteria

Toxicity		5-FU, Mitomycin C n = 35		Gemcitabine, Cisplatin n = 58	
	CTC-Grade		%		%
Leukocytes	2	9	26	23	40
	3	11	31	26	45
	4	2	6	2	3
Platelets	2	4	11	13	22
	3	3	9	14	24
	4	4	11	7	12
Haemoglobin	2	5	14	31	53
	3	3	9	2	3
	4	0	-	0	-
Nausea	2	11	31	23	40
	3	7	20	5	9
	4	0	-	1	2
Vomiting	2	7	20	19	33
	3	5	14	3	5
	4	1	3	1	2
Diarrhoea	2	2	6	4	7
	3	0	-	1	2
	4	0	-	0	-

## Discussion

Currently it is not clear which type of concurrent chemotherapy is best when combined with radiotherapy. While the standard of care for CRT is to combine a fluoropyrimidine with radiotherapy (5-fluorouracil or more recently also capecitabine) [10], there is a tendency to use more and more gemcitabine based chemoradiotherapy. The combination of 5-FU and mitomycin C that we have used in this report was previously employed for CRT in a number of trials in PDAC [22,23,32-34]. Mitomycin C was hypothesised to be useful in addition to 5-fluorouracil because of its predominant effectiveness in hypoxic conditions [35] since severe hypoxia was shown to be present in pancreatic tumours [36]. However, after the publication of a randomised phase III trial by Burris et al. [11] showing the superiority of gemcitabine compared with fluorouracil for the treatment of patients with advanced pancreatic cancer, much effort has been made to combine gemcitabine-based regimens concurrently with radiotherapy. This is reflected by the fact that during the last decade a total number of 36 clinical trials using gemcitabine and chemoradiotherapy have been published in PubMed, the majority of them after 2005. The combination of gemcitabine and cisplatin concurrently with radiotherapy which we have used in this analysis was also tested in a number of CRT trials [37-40] and chemotherapy trials [41]. Preclinical studies have suggested a synergistic interaction between gemcitabine cisplatin being the result of gemcitabine incorporation into DNA and an increase of platinum -DNA adduct formation [16,17]. Therefore, this study reports the use of two chemotherapeutic regimens based on biological hypotheses.

One of the strengths of this retrospective comparison is the homogeneity of the treatment variables and of the selection process of the patients for definitive chemoradiotherapy within one single centre in a large number of patients. Both, median overall survival time (12.7 versus 9.7 months) and 12-month overall survival rate (53% versus 40%) were statistically significantly longer in the patient cohort treated with the GC regimen compared to the FM regimen. Comparing our survival results with other trials which have investigated the use of 5-fluorouracil versus gemcitabine chemoradiotherapy, Crane et al. showed a trend favouring gemcitabine (53 vs 61 patients) based CRT [42] and Li et al. reported a statistically significant survival advantage for patients treated with gemcitabine concurrently to radiotherapy over those treated with 5-fluorouracil (16 vs 18 patients) [43]. In contrast, no advantage of gemcitabine over 5-fluorouracil CRT was detected in two other trials. However, the trial reported by Brasuniene et al. was very small (10 vs 9 patients per arm) and therefore was substantially underpowered to be able to detect any difference [44]. The second negative trial, reported by Wilkowski et al. [37] compared 3 arms, 5-fluorouracil (30 patients), GC (31 patients) and GC chemoradiotherapy followed by GC chemotherapy (27 patients). As this trial is the only one using the GC combination as the here reported trial, it is worth to compare the two trials in more detail. The median overall survival rate for the GC arm and the arm with GC CRT followed by GC chemotherapy was 9.3 and 7.3 months, respectively in this study whereas we observed a median overall survival rate of 12.7 months. A hypothetical explanation for this difference is a higher total radiation dose to the primary tumour in our trial (55.8 Gy versus 50 Gy). The patient characteristics between the two studies were similar. However, it needs to be stressed that our analysis is retrospective in nature and therefore we cannot exclude factors such as selection bias or other inhomogeneities. We have tested known factors influencing survival as good as possible and these included TNM staging and performance status and did not observe any significant differences. Just very recently a meta-analysis on the use of gemcitabine based chemoradiotherapy compared to 5-FU including 229 patients from randomised controlled trials was published [25]. This analysis described a survival advantage of gemcitabine based chemoradiotherapy compared to 5-Fu based for 12 month overall survival rates (RR 1.54, 95% CI 1.05 - 2.26, p = 0.03).

The toxicity analysis of the two regimens showed that the GC regimen led to a higher number of haematologic grade 3 toxicities, but interestingly not of grade 4 toxicities. Nausea and vomiting were the most frequent higher grade non-haematologic toxicities in both groups. Surprisingly, grade 3 nausea and vomiting were more frequent in the FM regimen despite of the emetogenic effect of cisplatin in the GC regimen. This might be attributable to the fact that antiemetic therapy has improved over time and FM being chronologically the first regimen used in this cohort. Comparing haematologic toxicity of the GC regimen in our trial with that reported by Wilkowski combined grade 3/4 leukocytopenia was comparable (48% versus 52%), grade 3/4 thrombocytopenia was less frequent in our trial (36% versus 52%) and grade 3/4 nausea was comparable (11% versus 13%). The addition of mitomycin C to 5-fluorouracil in our trial led to significant differences in haematologic grade 3/4 toxicity when comparing it with the Munich trial (thrombocytopenia: 20% versus 4%; leukocytopenia: 37% versus 4%) and nausea (20% vs 0%). The comparison of our study with the FFCD-SFRO [9] and with the ECOG [6] trials shows that our GC protocol resulted in lower GI toxicity compared to the ECOG regimen but more neutropenia which we attributed to the addition of cisplatin to gemcitabine. Compared with the FFCD trial our FM regimen led to a lower rate of non-haematological toxicity but a higher rate of thrombocytopenia which we attributed to the use of mitomycin C. The above mentioned recently published meta-analysis found significant differences of leukocytopenia, thrombocytopenia and gastrointestinal bleeding being more frequent in the gemcitabine group [25]. This might be due to suboptimal radiation techniques used in the trials with the majority of the patients being treated about a decade ago. At that time the toxicity-volume relationship of gemcitabine chemoradiotherapy was not yet described as well as now and IMRT was not yet as commonly used as it is now.

## Conclusions

Summarising our results in terms of efficacy and tolerance this retrospective report with its inherent limitations does not support the use of the FM regimen. Not only was it more toxic for grade 3 upper GI toxicity which is especially stressful for the patient but also less efficient and this is in line with a recent negative trial comparing chemoradiotherapy with radiotherapy only using this regimen [23]. On the other hand, the GC regimen was superior to FM in overall survival and reasonably well tolerated. However, tight upper limits of absolute treatment volumes have repeatedly described to be of high importance for the tolerance of gemcitabine based chemoradiotherapy regimens and we therefore advocate for very strict target volume definitions [27,45]. Last but not least it should be mentioned that currently the SCALOP trial in the UK compares gemcitabine vs. capecitabine based chemoradiotherapy in a randomised controlled phase II trial from which we expect will allow to draw firmer conclusions in the near future.

## Competing interests

The authors declare that they have no competing interests.

## Authors' contributions

TBB: Performed the retrospective analysis. TBB, RS, RF: developed the chemotherapeutic protocols. All the listed authors have been involved in drafting or in revising the manuscript. All authors read and approved the final manuscript.
